# Impact of Music on Working Memory in Rwanda

**DOI:** 10.3389/fpsyg.2020.00774

**Published:** 2020-04-28

**Authors:** Sara-Valérie Giroux, Serge Caparos, Nathalie Gosselin, Eugène Rutembesa, Isabelle Blanchette

**Affiliations:** ^1^Groupe de Recherche CogNAC, Département de Psychologie, Université du Québec à Trois-Rivières, Trois-Rivières, Québec, QC, Canada; ^2^DysCo Laboratory, Département de Psychologie, Université Paris 8, Saint-Denis, France; ^3^Institut Universitaire de France, Paris, France; ^4^International Laboratory for Brain, Music, and Sound Research, Université de Montréal, Montréal, Québec, QC, Canada; ^5^Center for Research on Brain, Language and Music, Université McGill, Montréal, Québec, QC, Canada; ^6^College of Medicine and Health Sciences, University of Rwanda, Kigali, Rwanda; ^7^École de Psychologie, Université Laval, Québec, Québec, QC, Canada

**Keywords:** music, working memory, Rwanda, trauma, cognition, arousal, valence, familiarity

## Abstract

Previous research shows that listening to pleasant, stimulating and familiar music is likely to improve working memory performance. The benefits of music on cognition have been widely studied in Western populations, but not in other cultures. The purpose of this study was to explore the impact of music on working memory in a non-Western sociocultural context: Rwanda. One hundred and nineteen participants were randomly assigned to a control group (short story) or one of four different musical conditions varying on two dimensions: arousal (relaxing, stimulating) and cultural origin (Western, Rwandan). Working memory was measured using a behavioral task, the n-back paradigm, before and after listening to music (or the short story in the control condition). Unlike in previous studies with Western samples, our results with this Rwandan sample did not show any positive effect of familiar, pleasant and stimulating music on working memory. Performance on the n-back task generally improved from pre to post, in all conditions, but this improvement was less important in participants who listened to familiar Rwandan music compared to those who listened to unfamiliar Western music or to a short story. The study highlights the importance of considering the sociocultural context in research examining the impact of music on cognition. Although different aspects of music are considered universal, there may be cultural differences that limit the generalization of certain effects of music on cognition or that modulate the characteristics that favor its beneficial impact.

## Introduction

Listening to music is a common activity, whether simply for the pleasure it promotes or for its psychological and cognitive benefits (Huron, 2007). The positive effect of music on emotion and stress regulation is well documented (Sloboda et al., 2001; Krout, 2007; DeNora, 2011; Chanda and Levitin, 2013; Hallam and MacDonald, 2013; Saarikallio et al., 2013; Habibi and Damasio, 2014). A beneficial effect of music on cognitive performance has also been reported. Studies have demonstrated higher performance on a number of cognitive tasks following exposure to music (Nantais and Schellenberg, 1999; Särkämö et al., 2008; Sutton and Lowis, 2008; Smith et al., 2010; Schellenberg and Weiss, 2013). This positive effect has been established for visuospatial tasks (Nantais and Schellenberg, 1999; Thompson et al., 2001; Husain et al., 2002; Schellenberg, 2005; Pietschnig et al., 2010), verbal tasks (Sutton and Lowis, 2008) and mathematical reasoning (Smith et al., 2010), speed of information processing (Schellenberg et al., 2007), creativity (Schellenberg et al., 2007), short-term visual memory (Chraif et al., 2014), and working memory (Mammarella et al., 2007; Chew et al., 2016; Palmiero et al., 2016). The vast majority of studies have focused on the effect of music on cognitive functioning in Western populations; very little research has investigated the effect of music in non-Western cultural contexts (Schellenberg et al., 2007; Chew et al., 2016). The purpose of this study was to examine the impact of music on working memory in an African culture, in Rwanda.

The literature also reports an alteration in cognitive performance related to traumatic exposure or highly stressful life events (Philip et al., 2013; Honzel et al., 2014; Scott et al., 2015). Trauma, as potentially experienced by a large part of the Rwandan population during the 1994 genocide perpetrated against the Tutsi, is therefore likely to negatively affect performance in certain cognitive tasks (Blanchette et al., 2019). Given the reports that music can have a positive effect on cognitive performance and emotion regulation, we examined whether this positive effect can be observed in Rwanda, a non-Western culture that could particularly benefit from it because of its widespread exposure to trauma.

The positive effect of music on cognition was first associated with Mozart’s music. In several studies, the performance of a group of students in a visuospatial task proved superior after they listened to Mozart’s music (Sonata for Two Pianos in D major, KV 448), compared to silence, a relaxation recording (Rauscher et al., 1993) or a short story (Rauscher et al., 1995). Subsequent research has found that the positive effect of music was not limited to Mozart’s compositions but could be observed with several types of music sharing certain characteristics (Nantais and Schellenberg, 1999).

Arousal is one of these characteristics (Thompson et al., 2001; Schellenberg, 2005). The arousal hypothesis states that listening to stimulating music characterized by fast tempo promotes a higher level of arousal among participants, which contributes to improved cognitive performance (Husain et al., 2002). One study manipulated the tempo of a musical excerpt to create a fast tempo version and a separate slow tempo version. Performance on a visuospatial task was superior after listening to music with fast tempo compared to music with slow tempo, supporting the arousal hypothesis (Husain et al., 2002).

Mood is another important characteristic associated with the effect of music on cognitive performance (Thompson et al., 2001). For example, the major mode, usually associated with happiness, favors the induction of a positive emotional state among participants (Dalla Bella et al., 2001; Gabrielsson and Juslin, 2003; Hunter et al., 2010). Music in major mode induces more positive mood which may also contribute to improving performance. In a study that manipulated the mode of a musical excerpt, participants who listened to the music in major mode showed a more positive mood, and better cognitive performance, than those who listened to music in minor mode (Husain et al., 2002). Music-induced mood changes is therefore another factor that can explain the cognitive improvement following music exposure.

In addition to arousal and mood, familiarity appears to be a third factor that can influence the effect of music exposure on cognitive performance. One study examined how background music affected academic performance and learning (Chew et al., 2016). Performance on a verbal memory task was significantly better when participants listened to music that was familiar to them, compared to unfamiliar music. Familiar music can also improve creativity (in drawing; Schellenberg et al., 2007).

In sum, music can improve cognitive performance (Nantais and Schellenberg, 1999; Nittono et al., 2000; Thompson et al., 2001; Sutton and Lowis, 2008), especially when it is familiar, when it promotes arousal, and when it induces a positive mood.

However, some of the results found in the literature are mixed. The positive effect of music is not always present or not always related to mood, arousal or familiarity. For example, in a study that used Mozart’s Sonata for two pianos in D major (a commonly-used piece; Thompson et al., 2001), working memory performance was less enhanced after listening to music than after a rest period (Kuschpel et al., 2015). Other data showed a beneficial effect of music on cognitive performance without, however, observing a link with self-reported arousal or mood (Smith et al., 2010). Another study looked at the effect of participants’ favorite music on their working memory performance (Hirokawa, 2004). Results indicated increased arousal when listening to music but no significant difference in working memory performance compared to other conditions: listening to relaxation instructions or remaining in silence.

These results conflict with those of studies showing a positive effect of music on cognition. These inconsistencies may however, largely be attributed to experimental procedures that vary from one study to another, particularly in regard to the difficulty of the cognitive task, the duration of music exposure, and the nature of the control group. The procedure in our study was modeled after the procedure used by Schellenberg and colleagues, as our hypotheses mainly rely on their work (see section “Materials and Method”).

The benefits of music for cognition have been widely studied in Western populations but very little in other cultures. Given the prominent place music occupies in all cultures (Peretz and Lidji, 2006; Munyaradzi and Zimidzi, 2012), it is important to test whether its effect on cognition is universal. In our study, we focused on Rwanda, an African country with a musical culture that is different from the Western one (see article by Munyaradzi and Zimidzi, 2012), and where music holds an important place (d’Ardenne and Kiyendeye, 2015).

The potential benefits of music may be particularly important to study in a country where a majority of individuals have been exposed to major trauma, during the 1994 genocide perpetrated against the Tutsis. One study has shown a negative link between the severity of traumatic experiences related to the genocide and short-term memory capacity, more than 20 years after the genocide (Blanchette et al., 2019). Another study showed impaired memory skills in Rwandan orphans, four years after the genocide (Dyregrov et al., 2000). Further, studies have documented that music has had a positive impact in reducing stress and anxiety in exposed populations in Rwanda (Walworth, 2003; Pelletier, 2004; Panteleeva et al., 2017; de Witte et al., 2019).

Studies of Western populations show that listening to music may improve cognitive performance, notably the working memory. In this study, we aimed to investigate the impact of music on working memory performance in a non-Western sociocultural context, Rwanda. We tested whether listening to music deemed familiar, pleasant and stimulating would enhance performance in a working-memory task.

## Materials and Methods

### Study Design

We studied the effect of music which varied according to two dimensions: origin (Western or Rwandan) and level of arousal (relaxing or stimulating). We measured working memory performance using an n-back task with two levels of difficulty (1-back or 2 back).

### Participants

One hundred and nineteen Rwandans participated in the study. Recruitment was done by Rwandan research assistants who verbally disseminated the information in different neighborhoods in Kigali (capital of Rwanda). Data collection took place over two three-week periods in July 2015 and August 2015. There were three inclusion criteria: (1) being at least 30 years of age, so that participants would have been old enough at the time of the genocide to remember the events; (2) having been present in Rwanda during the genocide; and (3) being able to speak and read Kinyarwanda. This last criterion was necessary because a large part of the study required reading questionnaires and task instructions on a computer screen. Seven participants had incomplete n-back measures (missing a data point in one of the four conditions), they were not included in the analyses. Participants who performed below the chance threshold (50%; *n* = 9) and those who performed worse in the 1-back condition (lower level of difficulty) than in the 2-back condition (higher level of difficulty; *n* = 11) were excluded from statistical analyses because of the possibility that they did not sufficiently understand the working memory task. The final sample included in the analyses therefore included 92 participants.

Participants were compensated 8,000 RWF, equivalent to approximately $ 15 CAD at the time of the study. An information letter was read to the participants, and they signed a consent form, before participating in the study.

### Procedure

This study was part of a larger research project exploring mental health, cognitive health and openness to reconciliation in Rwanda. Participants completed other questionnaires and cognitive tasks. The average total duration of the experimental procedure was approximately 3 h. The average duration of the tasks related to the study reported here was approximately 1 h.

The study was performed entirely on laptop computers. Questionnaires and tasks were presented using the E-Prime 2.0.10.353 software. Participants first answered a set of questionnaires aimed at documenting their mood and stress levels at the beginning of the task, as well as their degree of exposure to the genocide. They then completed the working memory task twice, before and after exposure to a sound excerpt (music or short story) which lasted 10 min. Participants wore headphones to listen to the sound excerpt. They were required to complete an assessment of their mood and arousal level before and after exposure to the sound excerpt. A subjective evaluation of the sound excerpt was performed at the end of the experiment.

At each moment, between four and seven participants were tested simultaneously, within the same room, each on a different computer. A doctoral student from the Université du Québec à Trois-Rivières (the first author) and one to two research assistants of Rwandan origin were present at all times to ensure the smooth running of the study, to provide clarification and information, and to assist participants with computer usage.

Participants were randomly assigned to one of five groups. Four experimental groups listened to music excerpts, which varied according to two dimensions: its origin (Western or Rwandan) and its effect on arousal (relaxing/slow tempo or stimulating/fast tempo). Participants in each group listened to musical excerpts which could be either Western and relaxing, Western and stimulating, Rwandan and relaxing or Rwandan and stimulating. Participants in the control group listened to a succession of two neutral short stories narrated in Kinyarwanda (one described the landscape seen on a trip to the mountains, the second described a child’s journey on the way to school).

### Material

#### Questionnaires

The questionnaires used in the study were translated from French or English to Kinyarwanda by two independent translators. The questionnaires were presented in the same order for all participants. For each question, participants selected a response by pressing the corresponding key on the numeric keypad. The possibility of not answering a question was always available by pressing the key “(9) I prefer not to answer.”

#### Profile of Mood States

A translated Kinyarwanda version of the Profile of Mood State Brief (POMS-B) questionnaire was used to measure participants’ mood. We used an abbreviated version of the original 65-item questionnaire (McNair et al., 1971). The POMS-B is a short version that contains 30 adjectives describing feelings and states of mood that the respondent may have experienced in the last week. This questionnaire is commonly used in cross-cultural studies. It has been validated and translated into several languages (Chen et al., 2002; Yeun and Shin-Park, 2006; Andrade et al., 2013). We used the three subscales of the POMS-B that were of interest to our hypotheses: Tension-Anxiety, Depression-Discouragement, and Vigor-Activity. Participants were asked to respond to items on a visual analog scale from *Not at all* to *Extremely*, in order to indicate the degree to which each adjective described their mood.

#### Perceived Stress Scale

The Perceived Stress Scale (PSS) measures the degree of stress associated with everyday life events (Cohen et al., 1983; Mimura and Griffiths, 2004; Cerclé et al., 2008). Participants responded to 14 items asking how often they had experienced a number of stress-related feelings or thoughts within the last month, using a Likert scale ranging from *Never* (0) to *Very often* (0). Total scores ranged from 0 to 56; a higher score corresponding to a higher level of stress. The PSS has good psychometric properties (Cohen et al., 1983) and is translated into 25 languages other than English (Lee, 2012). Given the absence of validated version in Kinyarwanda at the time of the study, we translated the English version for the present study.

#### Trauma Exposure

We assessed the severity of trauma exposure with the 9-item questionnaire used by Pham et al. (2004). Participants were asked if they had been exposed to potentially traumatic events related to the genocide: (1) damaged or lost property; (2) being forced to flee; (3) serious illness; (4) disability or illness resulting from the genocide; (5) sexual violence; (6) injuries to the body; (7) death of a close relative; (8) death of a close relative as a result of genocide-related illness; and (9) close relative who has been severely disabled as a result of the genocide. Participants were asked to answer each item with *Yes* (1) or *No* (0). The trauma exposure score ranged from 0 to 9. We included this questionnaire to control that the five groups were equivalent in terms of trauma exposure.

#### Sound Excerpts

The specific pieces of music used in the four conditions to compose the sound excerpts are presented in Table 1. The music of Western excerpts was instrumental. Considering the musical culture in Rwanda and the fact that Rwandan music is known to mostly contain lyrics, the Rwandan excerpts chosen for the study included lyrics. Finally, a combination of two short stories narrated in Kinyarwanda was presented to the control group.

**TABLE 1:** Titles of the sound excerpts.

Group		Sound excerpts
Origin	Arousal level	
Western	Relaxing	*Twenty Eight Parallel* (Vangelis)
		*Rousseau Meditation From Thais* (John Massenet)
		*Ave Maria* (Charles Gounod)
		*The Lord Bless you and keep you* (John Rutter)
Western	Stimulating	*Trumpet Concerto in E-Flat Major (Hob. VII e,1) III. Finale: Allegro* (Joseph Haydn)
		*Minute Waltz Op. 64 No. 1 in D flat* (Frédéric Chopin)
		*Rodondo Alla Turca* (Wolfgang Amadeus Mozart)
		*Dance of the Hours* (Amilcare Ponchielli)
Rwandan	Relaxing	*Umunezero* (Ceìcile Kayirebwa)
		*Kamaliza* (Mutamuliza Annonciata)
		*Rugamba* (Abeza Banjye)
Rwandan	Stimulating	*Ineza Y’Umuntu* (Theogene Uwiringiyimana)
		*Yakobo* (Iriba Choir)
		*Gutazira*
Short story		*Inkuru y’umwana w’umunyeshuri wibagiriwe ikayi mu rugo* (Dancille Mukarubibi)
		*Akarere k’iburasirazuba* (Dancille Mukarubibi)

*The composers appear in parentheses.*

All sound excerpts were normalized to a maximum amplitude of -1.0 decibels and faded in and out for 1 s, to avoid a startle effect and to obtain a gradual transition between silence and sound, and vice versa. The sound excerpts were presented using headphones for a duration of 10 min and the volume was previously adjusted to be comfortable (around 70 dB). At the end of the study, participants rated the valence, arousal level, and familiarity of the sound excerpt they had listened to, on scales ranging from 0 (very unpleasant, very relaxing, or not at all familiar) to 4 (very pleasant, very stimulating, or very familiar). The short stories were found to be neutral in terms of valence, activation and familiarity by participants in the control group.

#### Working Memory Task

**FIGURE 1:**
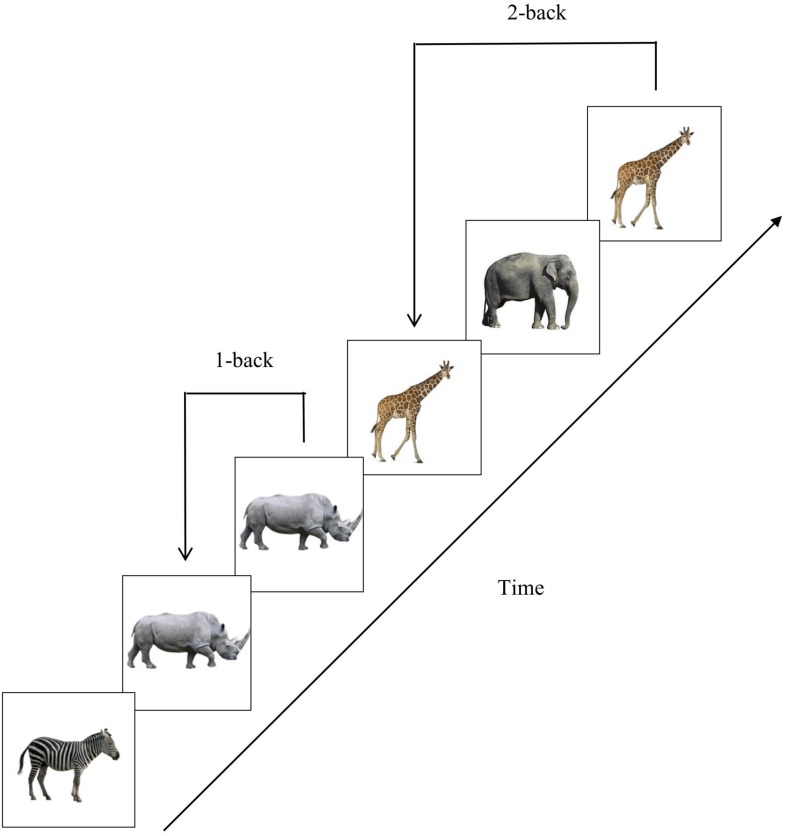
Representation of the procedure for the n-back task. Images of animals were presented successively. On each trial, participants had to indicate whether the image was the same as the one presented 1 or 2 items before.

After completing the questionnaires and before listening to the sound excerpt, participants performed an n-back task. In this task, a sequence of stimuli is presented on the screen. Participants must determine whether each stimulus is the same as the one presented *n* items earlier in the sequence. Figure 1 shows an example of stimuli sequences for 1-back and 2-back conditions. This task has been used to study working memory in both clinical and healthy populations (Blanchard et al., 1996; Rose and Ebmeier, 2006; Wild-Wall et al., 2011; Philip et al., 2016). This task requires monitoring, updating and manipulating information which is temporarily stored in working memory (Owen et al., 2005; Wild-Wall et al., 2011).

Considering the sociocultural context and the level of education of the sample, images of animals from the African continent, considered familiar stimuli, were used instead of the letters or numbers commonly used in n-back tasks (Schoofs et al., 2008; Schmiedek et al., 2014; Philip et al., 2016). Studies in the literature typically use 2-back and 3-back conditions (Schoofs et al., 2008; Lilienthal et al., 2013; Schmiedek et al., 2014). These studies include highly educated Western samples. We thus chose 1-back and 2-back conditions for our sample, which was on average, less educated.

Participants first completed two experimental blocks, one 1-back and one 2-back block, always starting with the 1-back one. After having listened to the 10-min sound excerpt (i.e., music or short story), they completed another two experimental blocks, again one 1-back and one 2-back. Each block consisted of 48 trials with a randomized presentation of the images. Each image was presented for 1 second, with two-second inter-stimulus intervals. Participants had to press one of two color-coded keys (green for “yes” or red for “no”) to indicate whether the stimulus presented on the screen was the same as the one presented n-trials earlier. Prior to the experimental blocks, detailed instructions with examples were given to ensure a good understanding of the task. Participants were also required to complete a practice block with 20 trials for each level of difficulty. Feedback was given indicating the number of correct and incorrect responses obtained on the practice block.

#### Self-Reported Psychological Measures

Before and after exposure to the sound excerpt, participants were asked to report their mood and their level of arousal on a Likert scale ranging from 0 to 4 (mood: *very sad* to *very happy*, and arousal level: *Very calm* to *Very excited*).

## Results

Statistical analyses were performed using the SPSS software. A first series of analyses was carried out to validate that the groups were equivalent, in terms of sex, age, and level of education, as well as in level of stress, mood and degree of trauma exposure (see Table 2 for descriptive statistics). The mean trauma exposure score for all participants was 4.42 (SD = 2.10) on the scale ranging from 0 to 9. An analysis of variance (ANOVA) was performed with group as a between-subject factor. There were no significant differences between groups, on age, perceived stress, mood and trauma exposure, *F*(4, 85) < 1.28, and *p* > 0.13. Gender and level of education also did not significantly differ between groups, *X*^2^ < 12.0, *p* > 0.20.

**TABLE 2:** Characteristics of participants according to the assigned group.

Group	Origin				
	Western	Western	Rwandan	Rwandan	Short story
		
	Arousal level				
	Relaxing	Stimulating	Relaxing	Stimulating	
**Sex**					
Men	*n* = 6	*n* = 12	*n* = 10	*n* = 8	*n* = 8
Women	*n* = 9	*n* = 10	*n* = 9	*n* = 10	*n* = 10
**Age**					
M (SD)	34.29 (4.76)	34.45 (7.24)	35.89 (7.96)	32.78 (2.37)	32.12 (1.69)
**Level of education**					
M (SD)	3.50 (0.76)	3.14 (1.06)	2.63 (0.83)	3.11 (0.96)	2.89 (0.83)
**PSS**					
M (SD)	30.00 (3.70)	30.59 (3.83)	34.32 (6.27)	30.06 (7.86)	31.06 (5.89)
**POMS Anxiety**					
M (SD)	6.79 (3.89)	6.00 (4.64)	7.16 (5.47)	5.50 (4.34)	4.83 (3.64)
**POMS Mood**					
M (SD)	6.64 (4.78)	5.24 (4.55)	6.74 (5.42)	4.94 (4.56)	4.72 (3.74)
**POMS Vigor**					
M (SD)	11.71 (3.05)	12.00 (2.30)	11.79 (3.97)	11.78 (4.25)	10.78 (3.37)
**Trauma exposure**					
M (SD)	4.87 (2.16)	4.38 (1.88)	4.32 (1.89)	4.33 (2.47)	4.28 (2.27)

*PSS, *Perceived Stress Scale*; POMS Anxiety, *Profile of Mood States* Tension-Anxiety scale; POMS Mood, *Profile of Mood States* Depression-Discouragement scale; POMS Vigor, *Profile of Mood States* Vigor-Activity scale.*

### Working Memory Performance

Analysis of variances were carried out to determine if performance on the n-back task differed according to difficulty level (1-back, 2-back), time (pre-, post-sound exposure), and group (Western relaxing music, Western stimulating music, Rwandan relaxing music, Rwandan stimulating music, control group). The ANOVA revealed a main effect of difficulty level, *F*(1, 87) = 222.46, *p* < 0.001, *n*^2^_p_ = 0.72 and time (pre, post), *F*(1, 87) = 26.57, *p* < 0.001, *n*^2^_p_ = 0.23. Participants performed better in the 1-back condition than in the 2-back condition, and performed better after sound exposure than before. There was also a marginally significant interaction between time and group, *F*(1, 87) = 2.19, *p* = 0.08, *n*^2^_p_ = 0.09. Difficulty level did not interact with group, *F*(4, 87) = 0.57, *p* = 0.69, *n*^2^_p_ = 0.02, time and difficulty did not interact, *F*(1, 87) = 0.49, *p* = 0.83, *n*^2^_p_ = 0.001, and the three way interaction was not significant, *F*(4, 87) = 0.31, *p* = 0.88, *n*^2^_p_ = 0.01.

To explore the contribution of the dimensions of music, a second ANOVA was performed, comparing the four groups who listened to music, excluding the 18 participants who listened to the narrated story. N-back performance (pre, post), averaged across 1-back and 2-back conditions, was compared according to the level of arousal of the music (relaxing or stimulating) and its origin (Western or Rwandan). The interaction between time and origin was significant, *F*(1, 70) = 7.35, *p* < 0.01, *n*^2^_p_ = 0.10. There was less improvement following music exposure when participants listened to Rwandan music (*M* = 0.01, SD = 0.08) compared to Western music (*M* = 0.06, SD = 0.08). No other effect was significant, *F*(1, 70) < 0.08, *p* > 0.78, excluding the main effect of time *F*(1, 70) = 16.46, *p* < 0.01.

**FIGURE 2:**
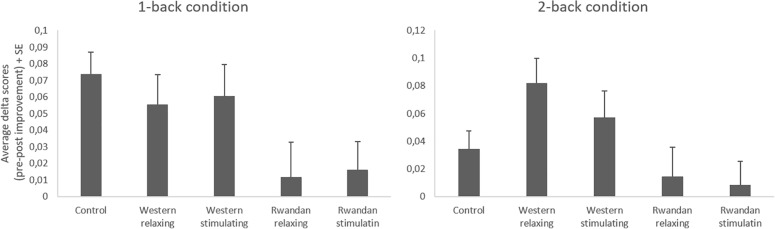
Average improvement (pre-post change score, with SE) in Working Memory performance in the different conditions after listening to one of four types of music (unfamiliar-Western vs familiar-Rwandan; stimulating vs relaxing) or a short story (control condition).

To examine the interaction in another way, we performed paired sample *t*-tests to compare performance pre and post-sound exposure, for each group separately (see Figure 2). A more conservative level of significance was used in these analyses (*p* < 0.01). These *post hoc* tests confirmed a significant improvement in working memory performance in the control group *t*(17) = 3.94, *p* < 0.001, and in the groups who listened to Western relaxing music, *t*(14) = 3.41, *p* < 0.001, or Western stimulating music, *t*(21) = 3.37, *p* < 0.001. The improvement was not significantly greater after listening to Western music than after listening to a narrated story (control condition), *t*(61) = 0.64, *p* = 0.52.

There was no significant improvement in the groups who listened to Rwandan relaxing music, *t*(18) = 0.76, *p* = 0.46 or Rwandan stimulating music, *t*(17) = 0.62, *p* = 0.55.

### Evaluation of the Musical Excerpts

Another series of analyses was carried out on participants’ evaluation of the musical excerpts. The mean evaluations of arousal, familiarity and valence are presented in Table 3. Three ANOVAs were performed, on each of these dependent measures, examining the effect of the level of arousal and the origin of the music as between-subjects variables. For arousal, there was a significant interaction between origin and level of arousal, *F*(1, 70) = 3.73, *p* = 0.05. *n*^2^_p_ = 0.05. Participants rated the Rwandan stimulating music as more arousing than the Rwandan relaxing music, *t*(35) = 2.28, *p* < 0.05, *d* = 0.94. The difference was not significant for Western music, *t*(35) = 0.49, *p* = 0.62, *d* = 0.16.

**TABLE 3:** Means (SD) of the evaluations of the musical excerpts.

Group	Origin			

	Western	Western	Rwandan	Rwandan
	
	Arousal level			
	Relaxing	Stimulating	Relaxing	Stimulating
Arousal level*	2.20 (1.32)	2.00 (1.15)	1.15 (0.93)	2.00 (1.41)
Familiarity*	1.07 (0.87)	1.24 (1.10)	2.95 (1.22)	3.33 (0.97)
Valence*	1.57 (1.14)	1.52 (1.19)	2.89 (0.88)	3.06 (0.87)

Standard deviations appear in parentheses. *As perceived by participants. Rating on a scale from 0 (minimum) to 4 (maximum).

Rwandan music was considered more familiar than Western music, *F*(1, 69) = 63.11, *p* < 0.001, *n*^2^_p_ = 0.48. Finally, Rwandan music was considered more pleasant than Western music, *F*(1, 69) = 33.23, *p* < 0.001, *n*^2^_p_ = 0.33. Rwandan music was therefore rated as more familiar and pleasant than Western music and the arousal level of stimulating Rwandan music was considered superior to relaxing music. No other effects were significant.

### Evaluation of Participants’ Mood and Level of Arousal

We analyzed participants’ self-assessments of mood and arousal before and after sound exposure using two ANOVAs (see Table 4). For mood, there was a significant interaction between time and group, *F*(4, 82) = 2.59, *p* = 0.04. Paired sample *t*-tests comparing self-reported mood pre and post-sound exposure showed a significant difference only in the group “Rwandan stimulating music,” *t*(15) = -3.10, *p* = 0.01. Participants in this group reported a happier mood after sound exposure. For arousal self-ratings, the interaction between time and group was not significant, *F*(4, 82) = 0.48, *p* = 0.75.

**TABLE 4:** Means (SD) of mood and arousal assessments by participants.

Group	Origin				

	Western	Western	Rwandan	Rwandan	Short story
		
	Arousal level				
	Relaxing	Stimulating	Relaxing	Stimulating	
**Mood**					
Pre	1.71 (1.31)	1.77 (1.19)	2.18 (1.07)	2.37 (1.09)	2.53 (1.13)
Post	2.00 (1.37)	2.24 (1.18)	2.35 (1.22)	3.00 (0.82)	2.83 (1.15)
**Arousal level**					
Pre	1.59 (1.18)	0.82 (0.59)	1.41 (0.62)	1.13 (0.50)	1.47 (0.80)
Post	1.06 (1.06)	1.05 (0.92)	1.35 (0.86)	0.87 (0.96)	1.28 (0.96)

*Standard deviations appear in parentheses. Pre: pre-sound exposure; Post: post-sound exposure. Rating on a scale from 0 (minimum) to 4 (maximum).*

## Discussion

This study aimed to explore the impact of music on working memory in a Rwandan sociocultural context. We explored the impact of music on working memory in a Rwandan population exposed to the 1994 genocide perpetrated against the Tutsi. Based on findings from studies of Western samples, our hypothesis was that listening to a familiar, pleasant and stimulating music would increase working memory performance.

Contrary to these predictions, our results do not show any positive effect of familiar, pleasant and stimulating music on working memory in this Rwandan sample. This contrasts with the improvement observed when participants were exposed to less familiar, Western music, or to a short story. This study does therefore not replicate results obtained in previous studies with Western samples, where familiar music was more likely to improve cognitive performance than unfamiliar music. Our results question the universality of the effect of familiarity. They suggest that, in non-Western populations, familiar music may not necessarily have a positive impact on cognitive performance, even when it is considered pleasant and stimulating, the two other features that have been shown to be associated with a beneficial impact of music on cognition.

Although our results appear to suggest that listening to Western music improved WM performance, this improvement was not greater than the one observed when participants listened to a narrated story. In all these conditions, participants’ performance improved with time and/or practice. This is noteworthy as it suggests that listening to familiar and pleasant music was in this case detrimental and masked an improvement that was otherwise observed. In this sense, listening to familiar and pleasant music interfered with performance.

Our results add to the conflicting evidence suggesting that the effect of music is not systematically observed, and may depend on the control condition that it is compared to, with effects being found more often when music is compared to silence, than to a control condition where a narrated story is presented (Hirokawa, 2004; Mammarella et al., 2007; Kuschpel et al., 2015; Chew et al., 2016; Palmiero et al., 2016). Furthermore, the potential interference effect of the control condition might differ depending on the task. In our study, we used a pictorial version of the n-back, instead of the letters or numbers that are typically used. All this needs to be considered when assessing the impact (or lack thereof) of Western music on working memory.

Although different aspects of music are considered universal, it is possible that the sociocultural context limits the generalization of certain effects of music on cognition, or at least modulates the characteristics that make music have an impact. The positive effect of music on cognitive performance has been demonstrated mainly in Western populations. Cognitive schemata related to music, such as melodic perception and emotional response, are likely, however, to be greatly influenced by culture (Gribenski, 2005; Demorest et al., 2008). The sociocultural context is important to consider when it comes to musical understanding and interpretation (Fiske and Royal, 2002). The results obtained in our study could be partly explained by the fact that cognitive schemata for musical information are culturally derived. If cognitive musical schemata differ between Rwandan culture and Western culture, the expected effects of music may also differ.

The Western and Rwandan excerpts induced similar levels of positive feelings and arousal, which are two important contributing factors. There are, however, many other important differences between the two types of music. The instruments featured in Rwandan music are different (including the ingoma, ikembe, and umuduri, unknown in Western music). Timbre and typical tempo may also differ. In our study, the Rwandan music contained lyrics while the Western music did not. However, the control group, who listened to a short story which also contained words, did show a significant improvement in working memory. Therefore, the lack of improvement with Rwandan music cannot be explained simply by the presence of lyrics. It is important to remember, however, that Western studies that show a positive effect on cognitive performance, for the most part, use music without lyrics. To determine whether features intrinsic to Rwandan music are responsible for the lack of effect, we will need to run additional studies, presenting the same excerpts to a Western sample.

It is important to highlight the methodological strengths of this study. First, the ratings of the selected music were entirely consistent with the intended type of each music. Rwandan music was evaluated as more familiar and pleasant than Western music. The arousal level of stimulating music from all origins was considered higher than that of relaxing music. Second, the n-back task showed the expected effect of level of difficulty. This confirms the validity of our adaptation of this working memory task, using images.

Future research should investigate more precisely the mechanisms responsible for the impact of music on cognitive performance. This would shed light on the specific reasons why the effect of music on cognition may differ depending on the sociocultural context, Western or African. It is important to better understand the cognitive and emotional processes involved in order to have a more accurate and informed understanding of the impact of music on cognitive performance. The role of cognitive resources in relation to music and cognitive performance should be investigated. The study of musical cognition from a cultural point of view will lead to a better understanding of the similarities and differences observed across cultures.

## Data Availability Statement

The raw data supporting the conclusions of this manuscript will be made available by the authors, without undue reservation, to any qualified researcher.

## Ethics Statement

The studies involving human participants were reviewed and approved, in Rwanda, by the National Ethics Committee of the Republic of Rwanda, the National Commission for Unity and Reconciliation and the National Commission for the Fight against Genocide. In Canada: the Ethics Committee of the Université du Québec à Trois-Rivières. The participants provided their written informed consent to participate in this study.

## Author Contributions

SG carried out the experiment under supervision from SC and IB. SG wrote the manuscript with support from IB, SC, NG, and ER. All authors provided critical feedback and helped shape the research, analysis and manuscript.

## Conflict of Interest

The authors declare that the research was conducted in the absence of any commercial or financial relationships that could be construed as a potential conflict of interest.
